# Attack Rate for Wild-Type SARS-CoV-2 during Air Travel: Results from 46 Flights Traced by German Health Authorities, January–March and June–August 2020

**DOI:** 10.1155/2022/8364666

**Published:** 2022-10-22

**Authors:** Felix Moek, Anna Rohde, Meike Schöll, Juliane Seidel, Jonathan H. J. Baum, Maria an der Heiden

**Affiliations:** ^1^Postgraduate Training for Applied Epidemiology (PAE), Department of Infectious Disease Epidemiology, Robert Koch Institute, Berlin, Germany; ^2^European Programme for Intervention Epidemiology Training (EPIET), European Centre for Disease Prevention and Control (ECDC), Stockholm, Sweden; ^3^Unit for Gastrointestinal Infections, Zoonoses and Tropical Infections (Unit 35), Department of Infectious Disease Epidemiology, Robert Koch Institute, Berlin, Germany; ^4^Unit for Crisis Management, Outbreak Investigations and Training Programmes, Focal Point for the Public Health Service (Unit 38), Department of Infectious Disease Epidemiology, Robert Koch Institute, Berlin, Germany

## Abstract

**Background:**

Evidence on the risk of SARS-CoV-2 transmission during air travel is scarce. We aimed to estimate the attack rate for wild-type SARS-CoV-2 to improve the evidence base for the adaptation of nonpharmaceutical intervention (NPI) strategies aboard airplanes.

**Methods:**

In collaboration with German Public Health Authorities (PHA), we conducted a follow-up of in-flight SARS-CoV-2 contact persons. We included those contact persons whom the Emergency Operations Centre at the Robert Koch-Institute had forwarded to PHA between January to March 2020 (before masking on flights became mandatory) and June to August 2020 (after the introduction of mandatory masking). We retrospectively collected data on whether these contact persons had been successfully contacted, had become symptomatic and had been tested for SARS-CoV-2, and whether alternative exposures other than the flight were known.

**Results:**

Complete data that allowed for the calculation of attack rates were available for 108 contact persons (median age of 36 (IQR 24–53), 40% female), traveling on 46 flights with a median flight duration of 3 hours (IQR 2–3.5). 62 of these persons travelled after masking on flights became mandatory. 13/87 developed symptoms, 44/77 were tested (no data for 21 and 31). 13 persons (9 of whom had been SARS-CoV-2 positive) were excluded from the analysis of attack rates due to a likely alternative exposure. We thus identified 4 probable in-flight transmissions (2 of which occurred after the introduction of mandatory masking). The overall attack rate resulted in 4.2% (4/95; 95% CI: 1.4%–11.0%). Considering flights after mandatory masking, the attack rate was 3.6% (2/56, 95% CI 0.6%–13.4%), before masking 5.1% (2/39, 95% CI 0.9%–18.6%).

**Conclusions:**

The risk of wild-type SARS-CoV-2 transmission during air travel seemed low, but not negligible. In order to formulate an effective, evidence-based NPI protocol for air travel, further studies considering the different transmissibility of SARS-CoV-2 variants of concern and vaccination status are needed.

## 1. Background

Since its discovery in December 2019 in China, the novel coronavirus SARS-CoV-2 has rapidly spread around the globe, accounting for numerous cases and deaths worldwide.

While there are many studies on SARS-CoV-2 attack rates in settings such as households, indoor recreational events, or travel on cruise ships, evidence on the likelihood of SARS-CoV-2 transmission during commercial flights is still scarce [1–4].

To our knowledge, the current literature on in-flight wild-type SARS-CoV-2 transmission largely consists of reports focusing on one or a few flights where transmission of SARS-CoV-2 has been suggested [5–23]. Only 3 studies were published that specifically reported the absence of transmission [12, 14, 21]. This suggests that the current body of evidence might be biased towards events where transmission has been documented, thus pointing to an overestimation of attack rates among flight contacts. Accordingly, a systematic review concluded that the quality of evidence of most published studies (up to January 2021) investigating in-flight attack rates was low [24]. Of the few systematic studies that have recently been published, none focused on time periods where masking during flights was mandatory [25, 26].

Here, we present the data of a systematic study of 46 flights where an in-flight exposure (i.e., a SARS-CoV-2 positive person during infectious period being on-board) has been documented, including flights where on-board masking was mandatory.

The goal of this study was to describe the attack rate among identified close flight contacts of individuals infected with wild-type SARS-CoV-2 (i.e., original Wuhan strain). This was done through a follow-up on flight-related SARS-CoV-2 contacts that were identified as part of the international SARS-CoV-2 contact tracing efforts at the Robert Koch Institute (RKI) in Berlin, Germany.

## 2. Methods

### 2.1. Study Design

We conducted a retrospective, cross-sectional study of prevalence of acute wild-type SARS-CoV-2 infection among close in-flight contact persons.

### 2.2. Contact Tracing of Flight Contacts at the Robert Koch Institute, Germany

As the national public health institute in Germany, the RKI distributes information between foreign countries and German public health authorities (PHA). For cross-border contact tracing during the COVID-19 pandemic, the RKI set up a specific international communication and contact tracing team (RKI IC-Team) [27].

Contact information for SARS-CoV-2 contact persons was forwarded through the federal state health authority to the respective PHA where the contact was living or staying at the time. These local PHAs then proceeded with contact tracing activities (i.e., telephone interview, regular monitoring) in accordance with the German Infection Protection Act (Infektionsschutzgesetz; IfSG).

#### 2.2.1. Definition of Contacts

Passengers within 2 rows from the index case were considered as high-risk contacts until 17 March 2020. This practice was based on recommendations of the World Health Organization for SARS-CoV-2 (28, 29). Between 18 March and 14 June, the RKI paused its recommendation to trace flight guests as the first wave hit Germany and PHA needed to prioritize their efforts.

On 15 June 2020, the recommendation on contact tracing of flight guests was resumed: direct seat neighbors were considered as high-risk and other persons within 2 rows as low-risk contacts.

#### 2.2.2. Definition of Primary Cases

A primary case was defined as a person with RT-PCR confirmed SARS-CoV-2 infection who had travelled by plane during the infectious period. This period was defined as 2 days before until 14 days after symptom onset for symptomatic individuals and 2 days before until 14 days after laboratory diagnosis for asymptomatic primary cases.

#### 2.2.3. Data Management

The RKI IC-team gave a cluster ID to each reported cross-border COVID-19 exposure event and archived them in a cluster list with access limited to its team members. This cluster list contained all available information relevant for contact tracing of the respective event. For flight-related clusters, these data included flight details, seat information of case/s and contacts, symptoms, date of symptom onset, and positive test result of the index case as well as contact details of close in-flight contacts.

### 2.3. Retrospective Follow-Up on COVID-19 Status of Contacts Successfully Traced by the German PHA

For this study, we reviewed the aforementioned cluster list to identify any contact persons for follow-up. We included contact persons for whom sufficient contact information was available and who could be assigned to a German PHA. Contact persons were excluded from follow-up when available information suggested any other exposure to SARS-CoV-2 in the 2 weeks prior to the flight. As an example, if it was known that passengers had shared hotels or restaurants or had been part of the same travel group as the index case, these clusters or specific contact persons within the clusters were excluded from follow-up.

In order to receive information on the SARS-CoV-2 status of identified contacts for this study, we contacted the German PHA that had received contact details for further follow-up during the acute event. The authorities were asked to review their records for SARS-CoV-2 related outcomes of the in-flight contacts. We asked whether authorities had been in contact with the identified contact persons, the date of contact, whether the persons had become symptomatic within 14 days after the flight, the type of symptoms, whether and when SARS-CoV-2 testing had been performed, test results as well as whether alternative SARS-CoV-2 exposures other than the flight were known. For this study-related follow-up, personal data were exchanged using an encrypted exchange server (Cryptshare®).

#### 2.3.1. Case Definition for Secondary Cases

Considering a SARS-CoV-2 incubation period of 2–14 days, we defined a probable flight transmission as a PCR-confirmed contact person with symptom onset (or sampling date if symptom dates were missing) within 2–14 days after the flight, without any known alternative exposure to SARS-CoV-2 before or after the flight. When symptom onset or sample collection date occurred 2 days after the flight, the case definition was only met if the primary case was known to be symptomatic on the day of the flight.

### 2.4. Calculation of the Attack Rate

For the calculation of the in-flight attack rate, we included those contact persons where the PHA could provide documentation that contact tracing had been successful, i.e., the PHA had been in contact with and informed the contact persons about the exposure. Attack rates were calculated by dividing the number of identified secondary cases by all successfully traced contact persons, expressed in percent.

### 2.5. Time Periods under Study: The effect of Mask-Wearing on Attack Rates

In May 2020, i.e., during the period when contact tracing of flight contacts was paused at RKI, the 1st version of the COVID-19 Aviation Health Safety Protocol was released and masking on-board airplanes became mandatory [28]. For the purpose of this study, we assumed that most passengers did not exercise air travel specific infection control measures (e.g., face mask) until May 2020 but that most if not all did afterwards. Data on the actual use of face masks were not available. In order to evaluate the effects of masking on attack rates, we compared the COVID-19 attack rates of all contacts identified before (23 January 2020 to 17 March 2020) vs. those identified after (5 June 2020 to 10 August 2020) masking on-board airplanes had become mandatory. As mentioned above, all flights under the study took place during a time before the surgence of other SARS-CoV-2 variants of concern (VOC), most of which differ in their transmissibility from the wild-type, original Wuhan strain under investigation here.

### 2.6. Ethical Consent and Data Protection

This study was reviewed and approved of by the ethics committee of the Charité-Universitätsmedizin Berlin, Germany (no. EA2/243/20). Compliance with the GDPR has been checked by the data protection department of the RKI; the implementation of the study was approved by the institute's management.

### 2.7. Data Quality Assurance and Statistical Analysis

To ensure data quality, 2 members of the study team independently entered data and validated for 10% of all cluster records selected for follow-up. As continuous variables were not normally distributed, medians and interquartile ranges (±IQR) were calculated. Categorical variables were reported as counts and frequencies. Proportions were compared using Fisher's exact test and chi-square test of independence, for ordinal data Wilcoxon rank sum test was used.

Microsoft Excel was used for data management where manual compilation of information was required. R using RStudio (The R Foundation for Statistical Computing, Vienna, Austria, and RStudio, Boston, the United States of America) was used for cleaning, management, and analysis of datasets.

## 3. Results

**Figure 1:**
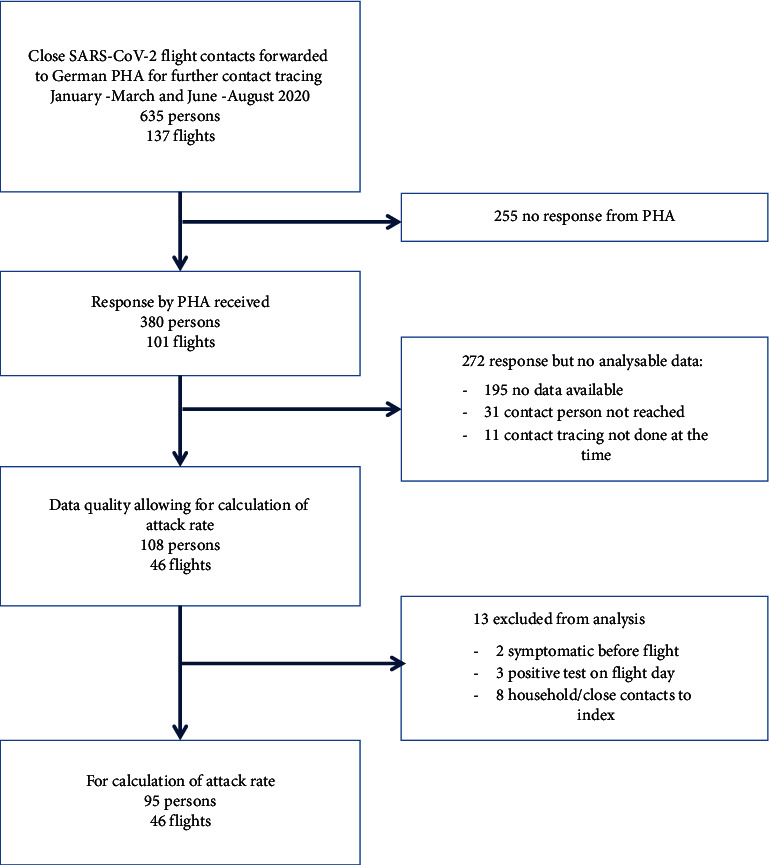
Flowchart for contact persons from follow-up with the PHA until inclusion in the calculation of attack rates, COVID-19 flight contact tracing study, Germany, 2020.

A total of 635 contact persons were identified for follow-up. These persons were distributed over 137 flights and 192 German PHA. PHA provided no feedback for 255 of 635 persons and, for an additional number of 272 persons, the available feedback was insufficient to calculate attack rates. 13 persons (among whom 9 were SARS-CoV-2 positive) were excluded from further analysis due to an alternative source of SARS-CoV-2 exposure as documented by the PHA. A flowchart from inclusion for follow-up until calculation of attack rates is depicted in Figure 1.

Thus, for the calculation of attack rates, we included 95 persons traveling on 46 flights. Detailed characteristics of all 95 persons, stratified by time periods before and after the introduction of masking, can be found in Table 1. The median age of contact persons and the proportion of symptomatic index cases differed significantly between the two time periods (*p*=0.01 and *p*=0.04, respectively). Notably, the median duration from detection of the index case to successful contact tracing reduced by one day between both time periods, possibly indicating an improvement in contact tracing efforts between and within countries.

Among the 95 persons described above, we identified 4 probable in-flight transmissions according to our case definition, 2 of which occurred before masking became mandatory (Table 2). All 4 probable in-flight transmissions were on flights with only one known index case.

The total attack rate amounted to 4.2% (95% CI 1.4%–11.0%). While the point estimate for the attack rate during the time period after the introduction of masking was slightly lower than before masking, confidence intervals overlapped widely (Table 3).

## 4. Conclusions

In this study among 95 in-flight contacts seated within two rows from the index case, we identified a total of 4 PCR-confirmed, probable in-flight wild-type SARS-CoV-2 transmissions.

Our results show that wild-type SARS-CoV-2 transmission also occurred during periods when masking on-board was mandatory. Although our results included time periods with and without masking, the low number of events did not allow for a meaningful statistical comparison of attack rates between periods.

To our knowledge, there are only a handful of studies to date that have investigated the in-flight attack rates for wild-type SARS-CoV-2 in a fashion comparable to ours, i.e., using the SARS-CoV-2 exposure (and not the outcome) as trigger for the study and including a large number of flights [22, 23, 25, 26]. This design has the advantage of not being overly biased towards flights where SARS-CoV-2 transmission has already been documented. Furthermore, so-called superspreading events among flight travels can be put into perspective as the calculated attack rates in this design represent the average of all included flights.

A study by Blomquist et al. that investigated flight contacts until March 2020 reported fairly similar attack rates of 3.8% (95% CI 1.3–10.6) among the 79 contact traced passengers sat within a 2-seat radius [25]. The comparable design, definition of cases, and close contacts probably contribute to these results being very similar to our findings [29].

An investigation by Hu et al. across 177 in-country flights departing from Wuhan before the lockdown on 23 January 2020 with a total of 5622 passengers resulted in a total attack rate of 0.6% (95% CI 0.43%–0.84%) [26]. This estimate, significantly lower compared to ours and to the results of Blomquist et al., might result from various reasons. In Wuhan, high-risk contacts included passengers within 3 rows seating distance, thus including 2 additional rows where transmission risk was possibly lower, thereby reducing the overall estimate. Moreover, the authors did not provide information on how many contact persons included for the calculation of attack rates had actually been successfully contact traced and/or tested. This might affect the estimate in at least 2 ways: if contact tracing/testing proportions were low, a significant number of in-flight transmissions might have gone undetected. On the other hand, if contact tracing was (nearly) complete, the estimate by Hu et al. would probably be more accurate and hereby highlight a limitation of our investigation, and that of Blomquist et al., both were retrospective analyses where only a minority of all in-flight contacts were successfully contact traced. Considering this, it is probable that infected contact persons were more likely to be successfully contact traced (e.g., as they might have sought testing even without the intervention by the PHA). Furthermore, records of COVID-19 cases were possibly more likely to still be available at the PHA when the retrospective data collection was done while those of negatively tested contact persons might already have been deleted. In both scenarios, the overall attack rate in our study would be biased towards higher estimates.

Most other limitations of our study are related to the fact that initial data collection was not done for study purposes but in order to facilitate fast and effective contact tracing. This might have triggered incomplete data on, e.g., symptom status of the index case or seating distance. However, we do not assume that these limitations significantly affected our results, since the local PHA usually validates whether a close contact had actually taken place. Also, although contact persons were often asked about alternative source of infection other than the flight and these persons were subsequently excluded from the analysis, we cannot completely rule out that community transmission might have affected our results.

We would like to point out that our investigation focused on in-flight transmission of wild-type SARS-CoV-2 and that conclusions on the attack rates of variants of concern (VOC) should only be made with great caution as most of these VOCs showed a different (and often increased) transmissibility when compared to the original Wuhan strain. To our knowledge, there are no studies that have investigated average attack rates over a greater number of flights for VOCs. In this regard, more research is direly needed.

As to the public health relevance of these findings, we believe that attack rates are one factor to be weighed in when prioritizing contact tracing of flight contacts against other efforts of disease control. Thus, depending as well on the public health infrastructure and capacities of each country, the decision on to what extent flight contacts are traced should also take other circumstances like the current disease epidemiology in the country of arrival, COVID-19 vaccine coverage and recent emergence of VOCs into consideration.

## Figures and Tables

**Table 1:** Characteristics of all contact persons included for the calculation of attack rates (*n* = 95), COVID-19 flight contact tracing study, Germany, 2020.

	Jan–Mar 2020 (no mandatory masking, *N* = 39^1^)	Jun–Aug 2020 (mandatory masking, *N* = 56^1^)	*p* value^2^	Total, *N* = 95^1^
Age	47 (31, 57)	36 (21, 49)	0.013	37 (24, 54)
No data	5	6		11
Sex			0.8	
Female	17 (44%)	19 (40%)		36 (42%)
Male	22 (56%)	28 (60%)		50 (58%)
No data	0	9		9
Developed symptoms after flight	5 (16%)	3 (6.7%)	0.3	8 (11%)
No data	8	11		19
Tested for SARS-CoV-2	12 (41%)	23 (59%)	0.2	35 (51%)
No data	10	17		27
Flight duration in minutes	140 (84, 245)	145 (112, 206)	>0.9	145 (100, 220)
Flight duration IATA categories			0.4	
Short-haul (<3 hours)	21 (54%)	37 (66%)		58 (61%)
Medium-haul (3–6 hours)	13 (33%)	15 (27%)		28 (29%)
Long-haul (6–16 hours)	5 (13%)	4 (7.1%)		9 (9.5%)
Index case was symptomatic	31 (100%)	38 (86%)	0.039	69 (92%)
No data	8	12		20
Symptom onset of index case			0.8	
Before flight	8 (26%)	8 (24%)		16 (25%)
On flight day	7 (23%)	10 (30%)		17 (27%)
After flight	16 (52%)	15 (45%)		31 (48%)
No data	8	23		31
No. of index persons per contact person			<0.001	
1	31 (79%)	49 (88%)		80 (84%)
2	1 (2.6%)	3 (5.4%)		4 (4.2%)
4	0 (0%)	4 (7.1%)		4 (4.2%)
7	7 (18%)	0 (0%)		7 (7.4%)
Seating distance to index case			0.017	
Direct seat neighbor	0 (0%)	5 (9.6%)		5 (5.8%)
Two rows	31 (91%)	47 (90%)		78 (91%)
Index case was cabin crew	3 (8.8%)	0 (0%)		3 (3.5%)
No data	5	4		9
No. of days from positive test of index to successful tracing	4 [3, 6]	3 [1, 4]	0.006	3 [3, 4]
No data	20	26		46
No. of days from flight to successful tracing	7.0 (4.0, 10.0)	6.0 (4.0, 9.0)	0.5	6.5 (4.0, 9.0)
No data	10	5		15

Median (IQR); *n* (%) Wilcoxon rank sum test; Fisher's exact test; Pearson's chi-squared test.

**Table 2:** Details of four probable in-flight transmissions, COVID-19 flight contact tracing study, Germany, 2020.

	Case 1	Case 2	Case 3	Case 4
Age	29	50	22	29
Sex	m	m	M	m
Developed symptoms after flight	Yes	Yes	Yes	Yes
No. of days from flight to symptom onset	2	5	11	3
Flight duration in minutes	115	420	125	80
Flight duration IATA categories	Short-haul (<3 hours)	Long-haul (6–16 hours)	Short-haul (<3 hours)	Short-haul (<3 hours
Index case was symptomatic	Yes	No data	No data	Yes
Symptom onset of index case	On flight day	NA	NA	After flight
Seating distance to index	Within 2 rows	One row before	One row behind	One row before

**Table 3:** Attack rates among close contact persons in total (*n* = 95), before (*n* = 39) and after (*n* = 56) masking, COVID-19 flight contact tracing study, Germany, 2020.

	Jan–Mar 2020 (no mandatory masking)	Jun–Aug 2020 (mandatory masking)	Overall
No cases/contact persons	2/39	2/56	4/95
Attack rate (95% CI)	5.1% (0.9%–18.6%)	3.6% (0.6%–13.4%)	4.2% (1.4%–11.0%)

## Data Availability

The underlying data can be available upon request through the corresponding author Felix Moek (felix.moek@gmail.com).
